# Socio-demographic factors, dental status, oral health knowledge and attitude, and health-related behaviors in dental visits among 12-year-old Shenzhen adolescents: a multilevel analysis

**DOI:** 10.1186/s12903-022-02110-8

**Published:** 2022-03-31

**Authors:** Jinfeng He, Bo Yuan, Shanyu Zhou, Shuyuan Peng, Ye Xu, He Cai, Li Cheng, Yuehua You, Tao Hu

**Affiliations:** 1 State Key Laboratory of Oral Diseases and National Clinical Research Center for Oral Diseases and Department of Preventive Dentistry West China Hospital of Stomatology, Sichuan University, Chengdu, China; 2 Department of Stomatology, Longhua People’s Hospital Affiliated to Southern Medical University, Shenzhen, 518109 Guangdong China; 3 School of Stomatology, Southern Medical University, Guangzhou, 510515 Guangdong China

**Keywords:** Adolescents, Cross-sectional study, Dental visits, Shenzhen, Oral health

## Abstract

**Background:**

Dental visits can provide education, prevention and treatment measures for teenagers, and help to form correct oral health knowledge and attitude. The purpose of this study was to evaluate the effects of socio-demographic factors, dental status, oral health literacy, and health-related behaviors on dental visits in early 12-year-old adolescents.

**Methods:**

953 subjects aged 12 in Longhua district of Shenzhen were investigated. The questionnaire and clinical examination were applied in schools, and two-level logistic regression models were constructed to interpret the effect of individual and contextual factors on Shenzhen adolescents' dental visits.

**Results:**

A total of 27.6% of the participants had not been to a dentist. After the multiple factors binary logistic regression analysis, it confirmed that the following variables: Shenzhen Hukou (OR 2.133, 95% CI 1.429–3.185), moderate caries (OR 1.404, 95% CI 1.022–1.928) and severe caries (OR 2.546, 95% CI 1.461–4.437), Angle Class II malocclusion (OR 1.703, 95% CI 1.134–2.556), sometimes or never toothbrushing (OR 2.985, 95% CI 1.491–5.975), dental floss usage (OR 1.829, 95% CI 1.250–2.677), having had a toothache within the last 12 months (OR 1.469, 95% CI 1.086–1.986), high knowledge attitude level (OR 1.570, 95% CI 1.106–2.229), moderate knowledge attitude level (OR 1.534, 95% CI 1.073–2.193), were associated factors for dental visit experience.

**Conclusions:**

The dental visits of 12-year-old children in Longhua district of Shenzhen is affected by multi-dimensional factors. It is suggested that oral health education should be strengthened, good oral hygiene habits should be cultivated, and the needs and utilization of oral health services for non-Shenzhen Hukou adolescents should be paid attention to, so as to effectively improve the overall oral health level of adolescents.

## Introduction

Dental caries is one of the most common oral diseases and the most important global oral health burdens with high prevalence around the world [1]. Dental caries is not only a childhood disease, but also can continue into adulthood [2], where health inequality still exists [3]. Adolescence is a transitional period from childhood to adulthood, an important milestone of physical, psychological and social transformation, it is also the key to the formation of oral health concepts and habits. Worldwide, the prevalence of dental caries in this stage ranged from 38.5 to 78% [1, 4, 5].

The availability and accessibility of oral health services are very important for the control of dental caries. Dental visits can provide education, prevention and treatment measures for teenagers, and help to form correct oral health knowledge and attitude. However, in developed countries, the rate of dental visits is 44%, and in developing countries, 18–38.3% of 12-year-old children have never seen a dentist or have not examed their teeth regularly [1]. The related factors affecting the dental visits of adolescents are complex, which need more attention.

Shenzhen, as a city with rapid economic growth, is also a city of immigrants. Immigrants describe individuals who migrate from their original living environment to other areas without local “Hukou” (China's population registration system), including the immigrant population from across provinces or rural areas to cities [6]. The number of children of migrant workers is huge, especially the Longhua district, which has a certain representativeness in economically and in geographically. When immigrants leave their original living environment and come into contact with urban consumption and lifestyle, their risk of non-communicable diseases increases [7]. At present, the oral health concept and behavior of the people in Longhua district are uneven and still in the stage of development and formation. Our pre survey and other articles have found that the prevalence of dental caries among 12-year-old adolescents in Longhua district is at a high level in all districts of Shenzhen, and is higher than that in the whole country [5, 8]. Therefore, we chose Longhua district to study the oral health status and medical resource utilization of adolescents under this special background.

The difference between China's population registration system and other countries is that it is not only aimed at determining personal identity, but also directly used to regulate population distribution and served for many other important objectives [9]. There are many situations to obtain Shenzhen Hukou, generally speaking, it represents a higher cultural level and better economic conditions. In China, public policies and social welfare programs have long been based on the “Hukou” system [6]. In Shenzhen, Hukou is still one of the main factors affecting the allocation of resources such as house purchase qualification and public education. The unfair distribution of resources brought by social development has always been the focus of the policymakers. Economic and sociological theories pay more and more attention to the coordination of social development and economic development. There are also more and more studies focus on the basic public health services utilization of migrants in China [10, 11]. In recent years, the equalization of public services has been promoting, so that the fruits of economic growth can be fully shared by the people. But there's much more to be done. To explore the dental treatment situation among different populations in Shenzhen will help to provide the government with the basis for oral health decision-making, eliminate the differences in oral health caused by the gap between the rich and the poor, and provide qualified labor force.

At present, the research on dental visits mainly focuses on sociodemographic factors, clinical factors and oral health literacy, etc. Sociodemographic factors are considered to influence the use of dental services through personal health behaviors. Family environment can affect the formation of oral health habits of children and adolescents, which was related to oral health behavior, eating habits and dental caries [12, 13]. Studies have reported the relationship between dental caries, toothache and the use of dental services by adolescents. We found an association between high oral health literacy and recent dental service use in adults, a higher level of occupational health helps to reduce inequalities in access to health care and dental information, which helps to visit dentists [14].

Oral health-related quality of life (OHRQoL) is an important indicator that can comprehensively reflect the impact of an individual's oral health problems or diseases on their physiological, psychological and social functions. Compared with objective indicators of oral examination, OHRQoL is a subjective indicator, which is an important part of patients' outcome report, including self-evaluation of oral health status, functional health, mental health, health service expectation and satisfaction and self-feeling [15]. In recent years, OHRQoL has been widely used in oral health survey and clinical research [16].

However, the impact of these factors on dental visits has not been fully explored in Chinese youth groups, especially in Shenzhen, one of the most characteristic cities. Exploring the relationships between dental visits and possible contributing factors may be beneficial for developing targeted interventions and healthcare policies. Therefore, the purpose of this study was to evaluate the effects of oral health literacy, sociodemographic, clinical and family characteristics on dental visits in early 12-year-old adolescents.

## Methods

### Study design

An analytical, cross-sectional study was conducted with 12-year-old students enrolled in public and private schools in the city of Shenzhen, China. Data collection was performed from 11/23/2020 to 11/27/2020. Ethics approval was obtained from Medical Ethics Committee of Longhua People’s Hospital Affiliated to Southern Medical University (Approval No. KY20201104).

### Survey sampling

Two-stage probabilistic cluster sampling was performed to select participants who were representative of the Longhua district of Shenzhen population. In the first stage, the number of private and public schools was selected. In the second stage, schools were randomly selected with probability proportional to size (PPS). The number of students included in the study was representative and proportional to the number of Shenzhen Hukou and non-Shenzhen Hukou students in the population of each district.

A letter of invitation and informed consent form were sent to the guardian of potential participants firstly, emphasizing that the participation was voluntary. Participants and their statutory guardians were required to sign informed consent forms. Adolescents with serious physical or psychological illness or disadvantages, who were unable or unwilling to complete the examination and questionnaire, were excluded. Ultimately, a random sample of students completed the survey; this number was greater than the expected participants calculated by the formula:

$$N = deff{*}\left( {\frac{{{\text{Z}}_{\alpha /2} }}{{\updelta }}} \right)^{2} \pi \left( {1 - \pi } \right)$$

in which the allowable error δ = 0.05 and α = 0.05 was adopted. π adopted the prevalence of gingival bleeding in people at aged 12 years from the Fourth National Oral Health Survey in China *p* = 58.4% [17]. A design effect of 2 was considered to correct for the sampling method, resulting in a sample of 747 adolescents. Then, 20% was added to compensate for expected missing data. The final sample was 934 adolescents. According to the class sampling survey of 100 students, a total of 10 schools needed to be selected.

### Quality control

Quality control was conducted as following: three licensed dentists, who had worked for more than 2 years and cooperated with three recorders, were trained by a standard examiner (the fourth examiner) before beginning this survey. The Cohen’s Kappa statistic was used to assess the inter-examiner variability of dental caries in adolescents and the final Kappa scores obtained on inter- or intra-examiner variability were ≥ 0.75. Furthermore, the inter-examiner variability in three regions during the survey were ≥ 0.75 as well.

### Questionnaire

The questionnaire was distributed to 1000 participants in 12 years old in Shenzhen.

All participants were asked to fill out the self-reported questionnaire, including demographic variables, socioeconomic status, dietary habit, oral health behavior, oral health-related knowledge, oral health-related attitude and the impact on oral health-related quality of life. The annual household income was not investigated because students may not be certain about it, and it was a sensitive issue. Therefore, the socioeconomic status here was tested by the economic situation of the street where the school is located and the parental educational level [18]. Parental educational level was classified into two levels: ≤ 8 years and > 8 years. Sugar intake level involved the consumption of desserts and candies, carbonated beverage (e.g., cola) and sugary drinks (e.g., sugary tea), which were clarified by five levels: Never, 1–3/month, 1/week, 2–6/week, 1/day, more than 2/day. These five levels were assigned with “Never” = 1, “1–3/month” = 2, “1/week” = 3, “2–6/week” = 4, “1/day” = 5, “More than 2/day” = 6 points, and then added the scores of 3 items, with the total score ranging from 3 to 18 points. The higher the total score, the more the sugar intake. The total score is divided into three grades according to the tri-sectional quantiles of accuracy. Oral health behavior included the frequency of tooth brushing, the frequency of dental flossing and the dental visit experience. The frequency of tooth brushing may choose more than once a day, once a day, less than once a day and never, then divided the participants into sometimes or never/daily. The frequency of dental flossing may choose daily, weekly, occasionally and never, then divided the participants into dental flossing usage /not. The dental visit experience was determined from the question ‘Did you ever go to the dentist?’ and clarified as yes and no. Oral health-related knowledge and attitude were evaluated by twelve questions about the understanding of the cause and prevention of oral diseases, the importance of oral health and the way to promote oral health. We rated each question with a score of 1 for correct answers, a score of 0 for wrong answers or unknown. We calculated the rate of correct answers and divided the participants into low, intermediate and high levels of knowledge and awareness according to the tri-sectional quantiles of accuracy [19]. The modified oral impact on daily performance (OIDP) scale was used to measure oral health-related quality of life (OHRQoL), which was widely used in the Fourth National Oral Health Survey in China. The scale included nine aspects: eating, pronunciation, brushing teeth or gargling, doing housework, going to school, sleeping, grinning, easy to worry and interpersonal communication. The items were simple and easy to understand, which was convenient for teenagers to make clear answers according to their own actual situation. As for the total score of 9 items in the OIDP scale, this study first assigned the options with “serious impact” = 1, “general impact” = 2, “slight impact” = 3, “no impact” = 4 points, and then added the scores of 9 items, with the total score ranging from 9 to 36 points. The lower the total score, the worse the oral health-related quality of life. The total score is divided into two grades. Set the total score > 27 as having no influence on oral health-related quality of life, and the total score ≤ 27 as having influence.

### Clinical assessment

All clinical procedures were conducted in the adolescents’ school in the following sequence. Each participant in the selected regions received an oral health examination by a trained licensed dentist according to the criteria issued by the WHO [20]. The portable equipment used consisted of a dental chair, external light source, flat dental mirror and the community periodontal index probe (WHO/CPI probe) in conjunction with WHO clinical criteria and visual examinations [20]. Dental caries status was assessed based on the methods and criteria recommended by the WHO [20]. The DMFT index was used to record the caries experience of permanent dentition [20]. Caries were detected mainly visually and recorded if a lesion had an unmistakable cavity, a shadow of discolored dentine visible through intact enamel, or a detectable softened floor or wall [20]. Signs of early caries, such as white or brown spot lesions and rough surfaces or fissures that were sticky to probing but without a detectable softened floor or wall, were not diagnosed as dental caries. Gingival bleeding was defined as the presence of gingival bleeding upon gentle probing (BOP) in at least one site [20]. The gingival calculus was explored by using CPI probe. The probe started just distal to the midpoint of the buccal surface and then gently moved into the mesial interproximal area. The same procedure was completed on the palatal surface. Bleeding sites were scored after the sites of a single quadrant were probed. Each site was scored as no bleeding = 0 and bleeding = 1. A gentle tactile exam was used to locate calculus deposits. Each site was scored as followings: no calculus = 0; calculus = 1. Probing depth, and clinical attachment level were not assessed. No radiographic examination was performed.

After completing the oral examination, all adolescents received an oral evaluation form to take home. This form classified the child’s oral health status according to the severity of the oral findings and recommended the timing of their next dental visit [20, 21].

### Statistical analysis

The data were independently extracted for the statistical analyses by two of the authors (H. C. and R. Z.). The whole information was extracted from the questionnaires and the oral health assessment form. EpiData Version 3.1 (EpiData Association, http://www.epidata.dk, Epidata Association, Odense, Den- mark) was used for data capture. In order to ensure the consistency and the accuracy of the data, the items were assessed more than 3 times during the input process. Any disagreements or mistakes were assessed further and dealt by the original data in questionnaires.

Descriptive analysis was performed, followed by bivariate and multivariate analyses using logistic regression analysis for complex samples. The dependent variable was dichotomized (yes/no) based on the adolescents’ reports of having dental visit experience. 10 Variables with a *P* value < 0.05 in the univariate bivariate analysis were incorporated into the multivariate analysis. Variables with a *P* value < 0.05 were maintained in the final model. Odds ratios (OR) and 95% confidence intervals (CI) were presented in both the bivariate and multivariate analysis. No multicollinearity problems were found among the variables in this study. The statistical procedures were performed in SPSS Statistics for Windows, version 22.0 (IBM Corp., Armonk, NY, USA).

## Results

A total of 1000 subjects aged 12 years were selected for eligibility in this study with an effective response rate of 95.3%. The final sample was composed of 953 12-year-old adolescents, 460 (48.3%) of whom attended public schools and 492 (51.7%) attended private schools. The non-response rate was 4.7%. Losses occurred due to successive absences from school on the days scheduled for the dental examinations (n = 21) and refusal to participate (n = 26). Among the participants, 49.1% (n = 468) of the participants were boys, and 50.9% (n = 485) were girls. A total of 27.6% of the participants had not been to a dentist within the last 12 months. The details of the total data were showed in Table 1.

**Table 1:** Descriptive characteristics of the participants (N = 953)

Variables	Number	Percent
Gender		
Male	468	49.1
Female	485	50.9
Hukou		
Shenzhen	227	23.8
Non-Shenzhen	726	76.2
Street economy		
Good	564	59.2
Poor	389	40.8
Father’s educational level		
≤ 8 years	407	42.7
> 8 years	546	57.3
Mother’s educational level		
≤ 8 years	481	50.5
> 8 years	472	49.5
Caries degree		
Zero	465	48.8
Moderate	370	38.8
Severe	118	12.4
Gingival bleeding		
No	248	26.0
Yes	705	74.0
Calculus		
No	597	62.6
Yes	356	37.4
Angle class II malocclusion		
No	767	80.5
Yes	186	19.5
Angle class III malocclusion		
No	730	76.6
Yes	223	23.4
Anterior deep overjet		
No	371	38.9
Yes	582	61.1
Toothbrushing frequency		
Daily	915	96.0
Sometimes or never	38	4.0
Dental floss usage		
No	714	74.9
Yes	239	25.1
Sugar intake level		
High	224	23.5
Moderate	606	63.6
Low	123	12.9
Toothache within the last 12 months		
No	468	49.1
Yes	485	50.9
Dentist visit experience		
No	263	27.6
Yes	690	72.4
Knowledge-attitude level		
High	306	32.1
Moderate	313	32.8
Low	334	35.0
Affect the OHRQoL		
No	247	25.9
Slight	614	64.4
Yes	92	9.7

In Table 2, the overall prevalence of dental caries was 51.2% (52.0% in Shenzhen Hukou and 51.0% in non-Shenzhen Hukou, respectively). The overall mean DMFT caries was 1.31 (1.33 and 1.30 in Shenzhen Hukou and non-Shenzhen Hukou, respectively). The filling rate was 26.9%, and this value was higher in Shenzhen Hukou (44.0%) than non-Shenzhen Hukou (21.4%). We define active caries as DT > 0 to describe the number of existing caries, the results showed that the prevalence of active caries in Shenzhen Hukou was 37.5%, which was lower than non-Shenzhen Hukou (45.0%). Then we found that the filling ratio of dental caries was different between Shenzhen Hukou and non-Shenzhen Hukou, so we were interested in the direct reason of the filling difference, and then led to logistic regression to explore the influencing factors of dental visit.

**Table 2:** Dental status in Longhua district of Shenzhen

	DMFT		Prevalence of dental caries (%)	Filling ratio (%)	Prevalence of active caries^a^ (%)
	${\overline{\text{x}}}$	S			
Hukou					
Shenzhen	1.33	1.84	52.0	44.0	37.5
Non-Shenzhen	1.30	1.80	51.0	21.4	45.0
Total	1.31	1.81	51.2	26.9	42.2

^a^Active caries means DT > 0

The training dataset characteristics and the results of univariate analyses were presented in Table 3, 10 variables in the univariate bivariate analysis were incorporated into the multivariate analysis. After the multiple factors binary logistic regression analysis (Table 3), the following variables were the risk predictors of dentist visit experience: Shenzhen Hukou 2.133 (1.429–3.185), moderate caries 1.404 (1.022–1.928) and severe caries 2.546 (1.461–4.437), Angle Class II malocclusion 1.703 (1.134–2.556), sometimes or never toothbrushing 2.985 (1.491–5.975), dental floss usage 1.829 (1.250–2.677), having had a toothache within the last 12 months 1.469 (1.086–1.986), high knowledge attitude level 1.570 (1.106–2.229) and moderate knowledge attitude level 1.534 (1.073–2.193). Sugar intake level and affecting the OHRQoL were not included in the final logistic regression model.

**Table 3:** Logistic regression for having dental visit experience and socio-demographic factors, dental status, oral health knowledge and attitude, and health-related behaviors

Variables	Dentist visit experience		Univariate analyses		Multivariate analyses	
	Yes	No				
	n (%)		*P* value	OR (95% CI)	*P* value	OR (95%CI)
Gender						
Male	347 (36.4%)	121 (12.7%)	0.237	1.187 (0.893–1.578)		
Female	343 (36.0%)	142 (14.9%)		1.00		
Hukou						
Shenzhen	190 (19.9%)	37 (3.9%)	**0.000**	**2.321(1.578–3.413)**	**0.000**	**2.133(1.429–3.185)**
Non-Shenzhen	500(52.5%)	226(23.7%)		1.00	1.00	
Street economy						
Good	462 (44.7%)	138 (14.5%)	**0.009**	**1.462(1.097–1.947)**		
Poor	264 (27.7%)	125 (13.1%)		1.00		
Father’s educational level						
> 8 years	406 (42.6%)	140 (14.7%)	0.118	1.256 (0.944–1.671)		
≤ 8 years	284 (29.8%)	123 (12.9%)		1.00		
Mother’s educational level						
> 8 years	360 (37.8%)	112 (11.8%)	**0.008**	**1.471 (1.104–1.959)**		
≤ 8 years	330 (34.6%)	151 (15.8%)		1.00		
Caries degree						
Severe	100 (10.5%)	18 (1.9%)	**0.000**	**2.698 (1.575–4.620)**	**0.001**	**2.546 (1.461–4.437)**
Moderate	277 (29.1%)	93 (9.8%)	**0.018**	**1.446 (1.067–1.961)**	**0.036**	**1.404 (1.022–1.928)**
Zero	313 (32.8%)	152 (15.9%)		1.00		1.00
Gingival bleeding						
Yes	519 (54.5%)	186 (19.5%)	0.158	1.256 (0.915–1.725)		
No	171 (17.9%)	77 (8.1%)		1.00		
Calculus						
Yes	247 (25.9%)	109 (11.4%)	0.108	0.788 (0.589–1.053)		
No	443 (46.5%)	154 (16.2%)		1.00		
Angle class II malocclusion						
Yes	148 (15.5%)	38 (4.0%)	**0.015**	**1.617 (1.096–2.385)**	**0.010**	**1.703 (1.134–2.556)**
No	542 (56.9%)	225 (23.6%)		1.00		1.00
Angle class III malocclusion						
Yes	150 (15.7%)	73 (7.7%)	**0.050**	**0.723 (0.522–1.001)**		
No	540 (56.7%)	190 (19.9%)		1.00		
Anterior deep overjet						
Yes	433 (45.4%)	149 (15.6%)	0.085	1.289 (0.966–1.720)		
No	257 (27.0%)	114 (12.0%)		1.00		
Toothbrushing frequency						
Sometimes or never	18 (1.9%)	20 (2.1%)	**0.001**	**3.073 (1.599–5.906)**	**0.002**	**2.985 (1.491–5.975)**
Daily	672 (70.5%)	243 (25.5%)		1.00		1.00
Dental floss usage						
Yes	196 (20.6%)	43 (4.5%)	**0.000**	**2.030 (1.407–2.928)**	**0.002**	**1.829 (1.250–2.677)**
No	494 (51.8%)	220 (23.1%)		1.00		1.00
Sugar intake level						
High	148 (15.5%)	76 (8.0%)	0.790	0.938 (0.588–1.498)		
Moderate	459 (48.2%)	147 (15.4%)	0.057	1.505 (0.988–2.291)		
Low	83 (8.7%)	40 (4.2%)		1.00		
Toothache within the last 12 months						
Yes	371 (38.9%)	114 (12.0%)	**0.004**	**1.520 (1.142–2.024)**	**0.013**	**1.469 (1.086–1.986)**
No	319 (33.5%)	149 (15.6%)		1.00		1.00
Knowledge-attitude level						
High	234 (24.6%)	72 (7.6%)	**0.007**	**1.618 (1.141–2.293)**	**0.012**	**1.570 (1.106–2.229)**
Moderate	233 (24.4%)	80 (8.4%)	**0.033**	**1.450 (1.031–2.039)**	**0.019**	**1.534 (1.073–2.193)**
Low	223 (23.4%)	111 (11.6%)		1.00		1.00
Affect the OHRQoL						
Yes	68 (7.1%)	24 (2.5%)	0.264	1.357 (0.794–1.891)		
Slight	455 (47.7%)	159 (16.7%)	0.055	1.371 (0.994–2.320)		
No	167 (17.5%)	80 (8.4%)		1.00		

Bold values denotes the risk predictors with *p* value < 0.05 and their respective OR (95% CI)

## Discussion

This study focused on the effects of socio-demographic factors, dental status, oral health literacy, and health-related behaviors on dental visits in early 12-year-old adolescents in Longhua district of Shenzhen. The probability proportional to size (PPS) sampling method was adopted in our study, and in the second stage of cluster sampling, we strictly followed the random method to select schools. All of these were to reduce selection bias and made the sample more representative of the subject. A clinical examination was conducted along with a self-reported questionnaire, including demographic variables, socioeconomic status, dietary habit, oral health behavior, oral health-related knowledge, oral health-related attitude and the impact on oral health-related quality of life. The overall prevalence of dental caries was 51.2%, which is consistent with the results of epidemiological survey in Guangdong Province in 2019 (57.83%) [8], and was much higher than the prevalence among 12-year-old children nationwide in China (38.5%) [5]. As an economically developed area, Longhua district of Shenzhen seems to have more serious dental caries, which will be discussed in our further epidemiological investigation. The mean DMFT caries of Shenzhen Hukou and non-Shenzhen Hukou are similar, which are 1.33 and 1.30 respectively, while there is a gap in the filling rate between them, which are 44.0% and 21.4%. These eventually lead to the difference in the prevalence of active caries, as the prevalence of DT > 0 in Shenzhen Hukou was 37.5%, which was lower than non-Shenzhen Hukou (45.0%).The results showed that treatment intervention could improve the status of active caries, and the reasons for the difference in filling rate between Shenzhen and non-Shenzhen accounts aroused our interest. Therefore, we further analyzed the dental visit and its influencing factors.

Multivariate binary logistic regression analysis found that, Shenzhen Hukou, more serious caries, Angle Class II malocclusion, without daily toothbrushing, dental floss usage, higher knowledge-attitude level might be the risk predictors of dental visit experience among 12-year-old adolescents. This is the first study on dental visit and related factors in Shenzhen's 12-year-old adolescents. These findings are important because they can help develop prevention strategies to reduce differences in the use of dental services during a period of changing oral health attitudes.

The dental visits probability of Shenzhen Hukou is twice that of non-Shenzhen Hukou. Shenzhen is one of the fastest growing cities in China, and its per capita GDP has been ranked first level in Chinese cities. The Longhua district has a large area of industrial zone, gathering a large number of migrant workers [22]. In this study, there are significant differences in dental visits between Shenzhen Hukou and non-Shenzhen Hukou, which reminds that the inequity of medical resources has been reflected in the oral diseases of 12-year-old children. At the same time of rapid economic construction, increasing investment in basic medical care may be something that policymakers need to pay more attention to. In particular, it is easier to improve the current situation of dental caries by promoting the oral health service utilization of non-Shenzhen Hukou (like community health care).

The dental visits probability of high caries is 2.5 times that of low caries, while the dental visits probability of middle caries is 1.4 times that of low caries, which indicates that the more serious caries is, the higher the dental visits probability is. Our results confirmed that of a previous study which found that need factors were always direct reasons for oral health service utilization [23]. The more serious the caries is, the more likely it is to see a dentist, similar findings have been reported [23, 24]. In addition, the continuous development of dental caries could further cause toothache, toothache experiences within the last 12 months were associated factors for dental visits, which was reported to be the most common reason for dental visits in most developing counties [25, 26]. Angle Class II malocclusion was also the reason to promote dental visit. It can be interpreted as Angle Class II malocclusion is handicapping both functionally and socially [27].

The dental visits probability of not-daily brushing is 2.98 times that of daily brushing. Not-daily brushing may lead to worse oral conditions and more need to seek medical services. People who use dental floss are 1.8 times more likely to see a dentist than people who don't use dental floss. Due to the low popularity of dental floss in China, it is not a conventional way of oral cleaning. Flossing habits represent a better awareness of oral cleaning, pay more attention to oral health, and have a higher probability of dental visit. The dental visits probability of high and medium knowledge-attitude level are both 1.5 times that of low knowledge-attitude level. Good oral health knowledge and attitude can also promote the dental visits in the same way with dental floss. A similar finding has been reported for adults [28]. Without good oral cleaning habits, oral problems are more likely to occur, which increases the demand for dental care, which is consistent with common sense. A previous study found that adolescents who received dental information had a great frequency of visiting a dentist compared to those who did not receive such information [29]. A higher level of oral health knowledge and attitude is believed to favor the acquisition of oral health information and exert a positive influence on visiting a dentist. This information is important, as the use of well-organized dental services based on preventive care contributes to the maintenance of oral health, which is particularly important in populations with a high risk of oral health problems. It shows that the popularization of oral health care knowledge is very important. Increasing publicity, depth and breadth, cultivating good oral health knowledge and attitude, and promoting good oral health habits are conducive to oral health.

The assumption of sugar intake was that it would lead to more caries, resulting in more dental visits, but this assumption has not been verified in this study. It may be that this indirect relationship is relatively slight. Similarly, we assume that dental visits will be promoted when poor oral health affects oral related quality of life. This relationship has not been verified, possibly because the impact on oral related quality of life is mostly slight or no and not statistically significant.

There must be limitations in our research, such as more data collection on family background, especially economic background. In addition, the cross-sectional design does not enable the determination of cause-and-effect relations among the variables. The main outcome of the study (dental visits) was self-reported by the adolescents, which increases the risk of memory bias, it is necessary to improve the design of epidemiological investigation in the future.

## Conclusion

The dental visits of 12-year-old children in Longhua district of Shenzhen is affected by multi-dimensional factors. Shenzhen Hukou, good oral hygiene habits and correct oral knowledge and attitude have a higher dental visit rate. People with Angle Class II malocclusion would be more eager to seek dental care. These results indicate the need for paying more attention to the demand and utilization of oral health services of non-Shenzhen adolescents. Strengthening oral health education and cultivating good oral hygiene habits may be a feasible intervene to effectively improve the overall oral health level of adolescents.

## Data Availability

The datasets used and/or analysed during the current study are available from the corresponding author on reasonable request.

## Abbreviations

OHRQoL: Oral health-related quality of life
CSDH: Commission on social determinants of health
WHO: World Health Organization
PPS: Probability-proportional-to-size
BOP: Bleeding on probing
CPIprobe: Community periodontal index probe
SE: Standard error
OR: Odds ratio
GDP: Gross domestic product

## References

[1] Petersen PE, Bourgeois D, Ogawa H, Estupinan-Day S, Ndiaye C (2005). The global burden of oral diseases and risks to oral health. Bull World Health Organ.

[2] Broadbent JM, Thomson WM, Poulton R (2008). Trajectory patterns of dental caries experience in the permanent dentition to the fourth decade of life. J Dent Res.

[3] Pitts N, Amaechi B, Niederman R, Acevedo AM, Vianna R (2011). Global oral health inequalities: dental caries task group-research agenda. Adv Dent Res.

[4] Duan SY, Li M, Zhao JL, Yang HY, He JF (2021). A predictive nomogram: a cross-sectional study on a simple-to-use model for screening 12-year-old children for severe caries in middle schools. BMC Oral Health.

[5] Quan JK, Wang XZ, Sun XY, Yuan C, Liu XN (2018). Permanent teeth caries status of 12- to 15-year-olds in china: findings from the 4th national oral health survey. Chin J Dent Res.

[6] Shao S, Zhang H, Chen X, Xu X, Du J (2021). Health education services utilization and its determinants among migrants: a cross-sectional study in urban-rural fringe areas of Beijing, China. BMC Fam Pract.

[7] Salazar MA, Hu X (2016). Health and lifestyle changes among migrant workers in China: implications for the healthy migrant effect. Lancet Diabetes Endocrinol.

[8] Cheng YH, Liao Y, Chen DY, Wang Y, Wu Y (2019). Prevalence of dental caries and its association with body mass index among school-age children in Shenzhen, China. BMC Oral Health.

[9] Chan KW, Zhang L (1999). The Hukou system and rural-urban migration in China: processes and changes. China Q.

[10] Song XL, Zou GY, Chen W, Han SQ, Zou X (2017). Health service utilization of rural-to-urban migrants in Guangzhou, China: does employment status matter?. Trop Med Int Health.

[11] Zhao Q, Huang ZJ, Yang SJ, Pan J, Smith B (2012). The utilization of antenatal care among rural-to-urban migrant women in Shanghai: a hospital-based cross-sectional study. BMC Public Health.

[12] Listl S (2011). Family composition and children’s dental health behavior: evidence from Germany. J Public Health Dent.

[13] Ferreira LL, Brandão GA, Garcia G, Batista MJ, Costa Lda S (2013). Family cohesion associated with oral health, socioeconomic factors and health behavior. Cien Saude Colet.

[14] Horowitz AM, Kleinman DV (2012). Oral health literacy: a pathway to reducing oral health disparities in Maryland. J Public Health Dent.

[15] Sischo L, Broder HL (2011). Oral health-related quality of life: what, why, how and future implications. J Dent Res.

[16] Gilchrist F, Rodd H, Deery C, Marshman Z (2014). Assessment of the quality of measures of child oral health-related quality of life. BMC Oral Health.

[17] Chen X, Ye W, Zhan JY, Wang X, Tai BJ (2018). Periodontal status of Chinese adolescents: findings from the 4th national oral health survey. Chin J Dent Res.

[18] Carmo CDS, Ribeiro MRC, Teixeira JXP, Alves CMC, Franco MM (2018). Added sugar consumption and chronic oral disease burden among adolescents in Brazil. J Dent Res.

[19] Wang L, Cheng L, Yuan B, Hong X, Hu T (2017). Association between socio-economic status and dental caries in elderly people in Sichuan Province, China: a cross-sectional study. BMJ Open.

[20] World Health Organization (WHO). Oral health surveys: basic methods. 5thed. Geneva: World Health Organization; 2013.

[21] Chen H, Zhang R, Cheng R, Xu T, Zhang T (2020). Gingival bleeding and calculus among 12-year-old Chinese adolescents: a multilevel analysis. BMC Oral Health.

[22] Zhou C, Gao Y, Wang S (2016). The characters and influencing mechanism of spatial-temporal variations of migrant workers in Shenzhen. Sci Geogr Sin.

[23] Xu M, Yuan C, Sun X, Cheng M, Xie Y (2018). Oral health service utilization patterns among preschool children in Beijing, China. BMC Oral Health.

[24] Qiu RM, Tao Y, Zhou Y, Zhi QH, Lin HC (2016). The relationship between children’s oral health-related behaviors and their caregiver's social support. BMC Oral Health.

[25] Machry RV, Tuchtenhagen S, Agostini BA, da Silva Teixeira CR, Piovesan C, Mendes FM, Ardenghi TM (2013). Socioeconomic and psychosocial predictors of dental healthcare use among Brazilian preschool children. BMC Oral Health.

[26] Villalobos-Rodelo JJ, Medina-Solís CE, Maupomé G, Lamadrid-Figueroa H, Casanova-Rosado AJ (2010). Dental needs and socioeconomic status associated with utilization of dental services in the presence of dental pain: a case-control study in children. J Orofac Pain.

[27] Dann C, Phillips C, Broder HL, Tulloch JF (1995). Self-concept, Class II malocclusion, and early treatment. Angle Orthod.

[28] Sistani MM, Yazdani R, Virtanen J, Pakdaman A, Murtomaa H (2013). Oral health literacy and information sources among adults in Tehran, Iran. Community Dent Health.

[29] Ekanayake L, Ando Y, Miyazaki H (2001). Patterns and factors affecting dental utilisation among adolescents in Sri Lanka. Int Dent J.

